# Genome-wide characterization of the GRF family and their roles in response to salt stress in *Gossypium*

**DOI:** 10.1186/s12864-020-06986-0

**Published:** 2020-08-24

**Authors:** Jun-Feng Cao, Jin-Quan Huang, Xia Liu, Chao-Chen Huang, Zi-Shou Zheng, Xiu-Fang Zhang, Xiao-Xia Shangguan, Ling-Jian Wang, Yu-Gao Zhang, Jonathan F. Wendel, Corrinne E. Grover, Zhi-Wen Chen

**Affiliations:** 1 National Key Laboratory of Plant Molecular Genetics and National Center for Plant Gene Research, Institute of Plant Physiology and Ecology/CAS Center for Excellence in Molecular Plant Sciences, Chinese Academy of Sciences, Shanghai, 200032 China; 2 Plant Stress Biology Center, Institute of Plant Physiology and Ecology/CAS Center for Excellence in Molecular Plant Sciences, Chinese Academy of Sciences, Shanghai, 200032 China; 3 University of Chinese Academy of Sciences, Shanghai, 200032 China; 4 Esquel Group, 25 Harbour Road, Wanchai, Hong Kong, China; 5 School of Life Science and Technology, ShanghaiTech University, Shanghai, 201210 China; 6 Department of Ecology, Evolution and Organismal Biology, Iowa State University, Ames, IA 50011 USA; 7 Institute of Carbon Materials Science, Shanxi Datong University, Datong, 037009 China

**Keywords:** Growth regulating factor, Cotton, Phylogenetics, Organ-specific expression, Salt stress

## Abstract

**Background:**

Cotton (*Gossypium* spp.) is the most important world-wide fiber crop but salt stress limits cotton production in coastal and other areas. Growth regulation factors (GRFs) play regulatory roles in response to salt stress, but their roles have not been studied in cotton under salt stress.

**Results:**

We identified 19 GRF genes in *G. raimondii*, 18 in *G. arboreum*, 34 in *G. hirsutum* and 45 in *G. barbadense*, respectively. These GRF genes were phylogenetically analyzed leading to the recognition of seven GRF clades. GRF genes from diploid cottons (*G. raimondii* and *G. arboreum*) were largely retained in allopolyploid cotton, with subsequent gene expansion in *G. barbadense* relative to *G. hirsutum*. Most *G. hirsutum* GRF (*GhGRF*) genes are preferentially expressed in young and growing tissues. To explore their possible role in salt stress, we used qRT-PCR to study expression responses to NaCl treatment, showing that five *GhGRF* genes were down-regulated in leaves. RNA-seq experiments showed that seven *GhGRF* genes exhibited decreased expression in leaves under NaCl treatment, three of which (*GhGRF3*, *GhGRF4*, and *GhGRF16*) were identified by both RNA-seq and qRT-PCR. We also identified six and three *GRF* genes that exhibit decreased expression under salt stress in *G. arboreum* and *G. barbadense*, respectively. Consistent with its lack of leaf withering or yellowing under the salt treatment conditions, *G. arboreum* had better salt tolerance than *G. hirsutum* and *G. barbadense*. Our results suggest that GRF genes are involved in salt stress responses in *Gossypium*.

**Conclusion:**

In summary, we identified candidate GRF genes that were involved in salt stress responses in cotton.

## Background

Cotton (from the genus *Gossypium*) is the most important fiber crop in the world. While the genus itself contains more than 50 species, cultivated cottons are composed of four independently domesticated species, among which the Upland cotton (*G. hirsutum* L.) is the most prominent cultivar, accounting for 95% of global cotton fiber output [1, 2]. Due to the agronomic importance of cotton, it is worth studying its defense and stress responses. Cotton plants produce gossypol and related sesquiterpene aldehydes that function as defensive compounds against pests and pathogens [3–7]. With respect to abiotic stresses, cotton has a moderate tolerance to salinity and consequently is cultivated in some saline-alkali soils; however, salt stress is still a limiting factor that affects cotton production [8].

Identification and characterization of salt response genes is important in dissecting the molecular mechanisms of plant adaptation to salt stresses and, ultimately, in engineering salt-tolerant crops. Consequently, research into the genetic responses to salt stress has yielded much information (see recent reviews, such as [9–12]), including the role of growth regulation on salt tolerance. Growth-regulating factors (GRFs) are plant-specific transcription factors, which form a relatively small family and function in plant development and stress responses [13]. The first GRF was identified from rice, which uncovered a regulatory role for GRFs during leaf and stem development [14]. Subsequent research showed that these transcription factors are also involved in other aspects of plant growth and adaptation, including root development [15], flowering [16, 17], leaf size and longevity [18], and response to abiotic stresses [19–22]. Recent research on abiotic stress responses have reported a role for GRF repression in abiotic stress tolerance. For example, the activation of the *Arabidopsis* stress-responsive gene AtDREB2A, whose expression increases plant tolerance to osmotic stress [23, 24], requires repression of at least one GRF. Under non-stress conditions, AtGRF7 binds to the *DREB2A* promoter to suppress expression; however, abiotic stress leads to suppression of AtGRF7 expression and consequently the activation of osmotic stress-responsive genes [20].

Recent advances in cotton genomics have produced the resources necessary to characterize the GRF gene family in *Gossypium.* Multiple high-quality genome sequences are available for several species, including two diploid species, i.e., *G. raimondii* (D_5_) [25, 26] and *G. arboreum* (A_2_; cultivated) [27, 28], and two cultivated allotetraploids, namely *G. hirsutum* (AD_1_) [29–31] and *G. barbadense* (AD_2_) [31–34]. These resources provide the foundation for identifying the suite of GRF genes in *Gossypium* and their potential roles as salt stress-related genes. Here, we report our genome-wide analysis of the GRF transcription factor genes in four important cotton species, reporting a modestly sized family (18 and 19 members in diploid species, 32 and 45 in polyploids) that are largely conserved during evolution and polyploidization. We further explore the role of GRF genes in response to salt stress. We identified three *G. hirsutum* GRF genes, six *G. arboreum* GRF genes, and three *G. barbadense* GRF genes that exhibit decreased expression under salt stress, respectively. Compared to the tetraploid species, diploid cotton *G. arboreum* was more salt tolerant. These candidate GRF genes may be useful in future molecular breeding of salt-tolerant cotton species.

## Results

### Genome-wide identification and sequence analysis of genes encoding putative GRFs in *G. hirsutum*

**Fig. 1:**
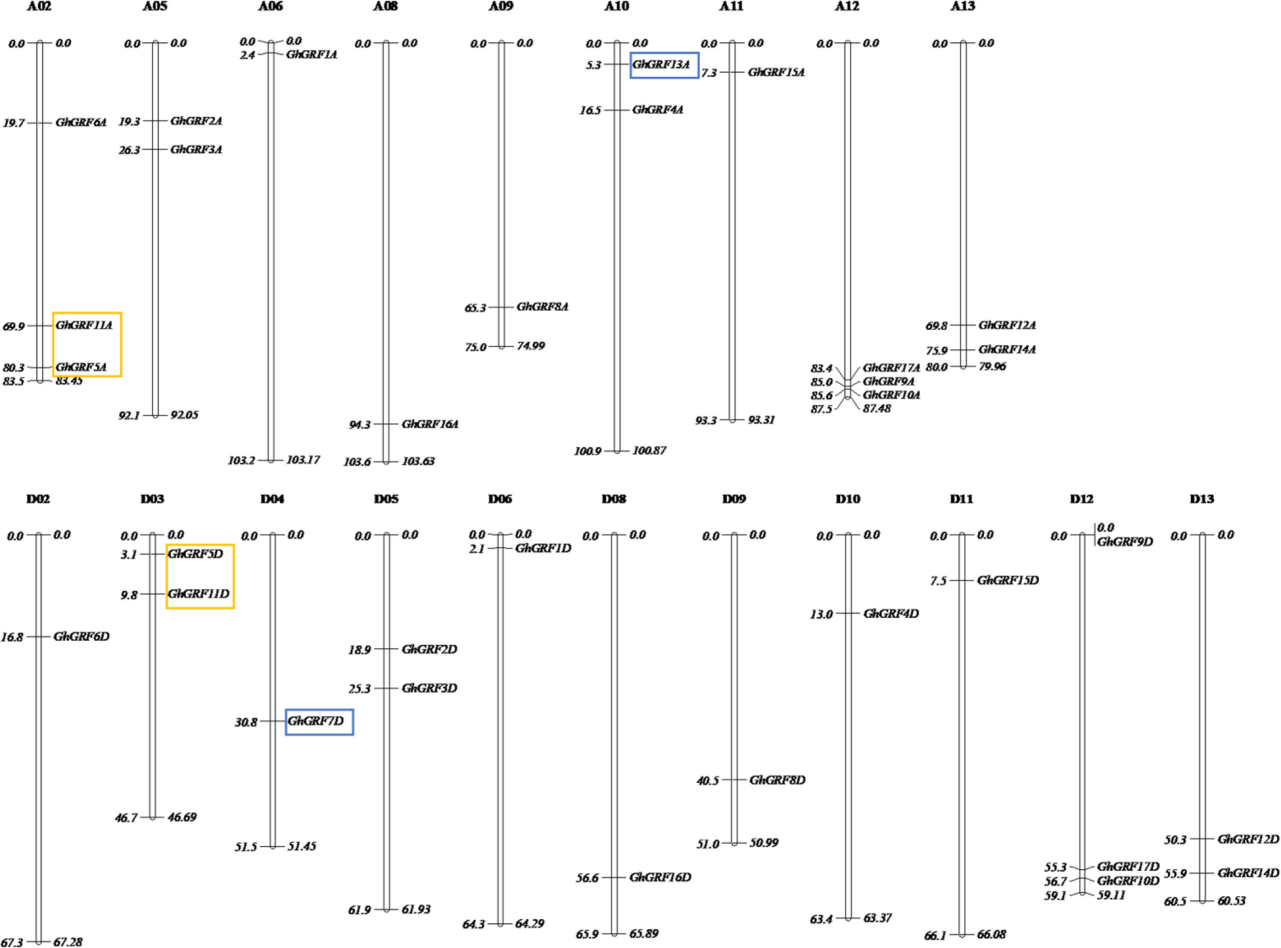
Dispersed distribution of GRF genes in *G. hirsutum* (AD_1_) chromosomes. 32 *GhGRF* genes are scattered over 20 of the 26 *G. hirsutum* chromosomes. Four genes in two yellow boxes are located in the A_t_ translocation regions between chromosomes A02 and A03. Two genes in two blue boxes represent the corresponding homoeologous genes undetectable in the other subgenome, respectively

GRF proteins are involved in plant responses to abiotic stresses [19, 22], including salt stress [20]. These proteins are defined by the presence of a QLQ domain for protein-protein interactions, a WRC domain responsible for DNA binding and a putative nuclear localization signal [13, 14, 35]. Hmmersearch against the *G. hirsutum* genome database with these two conserved domains (PF08879 for WRC domain and PF08880 for QLQ domain) identified 34 putative GRF-encoding genes, here designated *GhGRF1A*–*GhGRF17A* (A homoeologs) and *GhGRF1D*–*GhGRF17D* (D homoeologs), where the A and D suffix in the gene name indicates the genome of origin. These 34 *GhGRF* genes are dispersed over 20 of the 26 *G. hirsutum* chromosomes, with most, but not all, homoeologs conserved (Fig. 1). Syntenic conservation of two homoeologous pairs, i.e., *GhGRF5A/D* and *GhGRF11A/D*, was disrupted by a known large chromosomal translocation [29, 30, 32]. This translocation resulted in these conserved homoeologs being located on the non-homoeologous chromosomes A02 and D03 (Fig. 1). In addition, a single homoeolog each was recovered for both *GhGRF7D* and *GhGRF13A* (Fig. 1) by conserved domain search; however, BLAST recovered syntenic copies of GRF-like genes for both genes that could represent the missing homoeologs, i.e., *GhGRF7A* and *GhGRF13D*. Both syntenic GRF-like homoeologs lack the canonical conserved GRF protein domains, possibly indicating a loss of, or change in, function; therefore, these homoeologs were excluded from further analyses.

### Structural organization of *GhGRF* genes

**Fig. 2:**
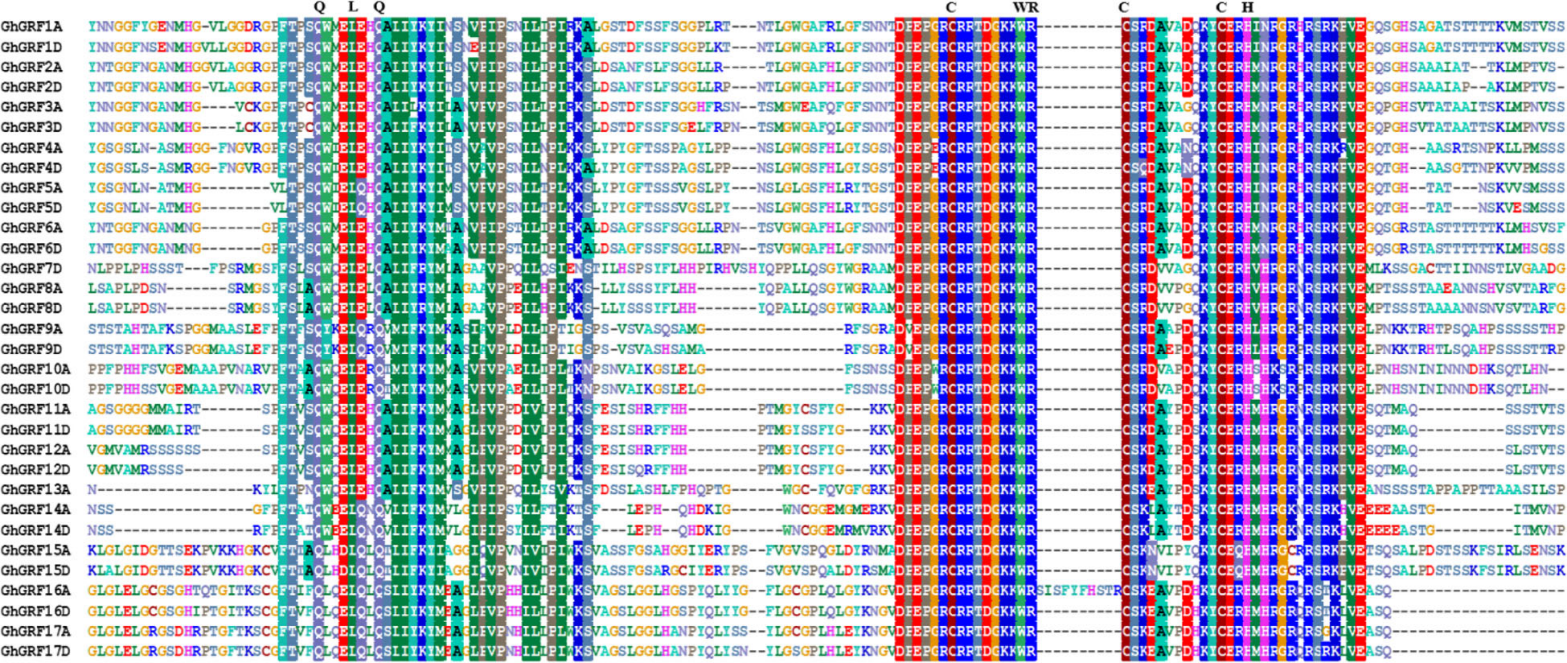
Sequence alignment of GhGRF (*Gossypium hirsutum* GRF) proteins and the QLQ and WRC domains are indicated upside. Identical amino acids are indicated by the color background

More than threefold variation in length was detected in the predicted coding sequences (CDS) for the recovered *GhGRF*s, from 546 bp for *GhGRF14A*/*D* to 1833 bp for *GhGRF2A*/*D*, (Table 1), which translates to proteins ranging from 181 amino acids (aa) (20.73 kDa) to 610 aa (65.58 kDa). Predicted isoelectric points (pI) for members of this family also vary widely, from 5.89 to 9.59, possibly due to the composition of the C-terminal region (Additional file 3: Figure S1). All of the putative GhGRF proteins have both QLQ and WRC domains [14] in the N-terminal region (Table 1, Fig. 2, Additional file 3: Figure S1). GhGRF15A and GhGRF15D also contain a second WRC domain downstream of the first one (Additional file 3: Fig. S1). A zinc finger motif (CCCH) was also observed within the WRC domain in all putative GhGRF proteins (Fig. 2).

**Table 1:** Sequence characteristics of *GhGRF* (*Gossypium hirsutum* growth-regulating factor) genes and corresponding proteins

Gene Name	Locus Name	Chr	Genomics position	CDS	No. of Introns	Size (aa)	QLQ Domain	WRC Domain	MW	pI
GhGRF1A	Gh_A06G0219	A06	2,427,674–2,430,873	1782	3	593	161–197	232–274	64.42	8.83
GhGRF1D	Gh_D06G0212	D06	2,081,549–2,084,687	1731	3	576	162–198	233–275	62.37	6.48
GhGRF2A	Gh_A05G1848	A05	19,322,416–19,325,837	1833	3	610	162–198	231–273	65.38	8.36
GhGRF2D	Gh_D05G2044	D05	18,868,989–18,872,409	1833	3	610	160–196	231–273	65.58	8.06
GhGRF3A	Gh_A05G2252	A05	26,265,366–26,267,943	1815	3	604	143–179	215–257	65.27	6.58
GhGRF3D	Gh_D05G2512	D05	25,255,757–25,258,332	1815	3	604	143–179	215–257	65.41	6.48
GhGRF4A	Gh_A10G0804	A10	16,451,418–16,453,809	1428	3	475	139–175	210–252	52.25	6.63
GhGRF4D	Gh_D10G0959	D10	12,983,506–12,985,893	1428	3	475	139–175	210–252	52.03	6.34
GhGRF5A	Gh_A02G1438	A02	80,257,374–80,259,722	1569	3	522	132–168	203–245	57.25	7.71
GhGRF5D	Gh_D03G0282	D03	3,085,927–3,089,051	1686	3	561	140–176	211–253	61.24	8.14
GhGRF6A	Gh_A02G0827	A02	19,707,965–19,710,598	1695	3	564	144–180	216–258	61.30	7.26
GhGRF6D	Gh_D02G0876	D02	16,810,024–16,812,651	1710	3	569	144–180	216–258	61.70	6.97
GhGRF7D	Gh_D04G1000	D04	30,836,475–30,838,388	1092	3	363	73–109	147–189	39.96	9.13
GhGRF8A	Gh_A09G1285	A09	65,252,506–65,254,792	1218	3	405	83–119	149–191	44.21	8.25
GhGRF8D	Gh_D09G1332	D09	40,520,770–40,523,059	1209	3	402	81–117	147–189	43.77	8.54
GhGRF9A	Gh_A12G2234	A12	85,044,073–85,048,399	1491	4	496	101–137	158–200	53.51	6.96
GhGRF9D	Gh_D12G2699	D12	7626–9481	1491	3	496	102–138	159–201	53.58	6.94
GhGRF10A	Gh_A12G2177	A12	84,589,570–84,591,240	1401	3	466	152–188	210–252	50.86	5.89
GhGRF10D	Gh_D12G2356	D12	56,690,643–56,692,281	1377	3	458	144–180	202–244	50.05	5.95
GhGRF11A	Gh_A02G1205	A02	69,876,127–69,879,623	1008	2	335	30–66	93–135	36.71	7.74
GhGRF11D	Gh_D03G0527	D03	9,819,619–9,823,083	1026	2	341	30–66	93–135	37.40	7.81
GhGRF12A	Gh_A13G1365	A13	69,805,651–69,809,400	1005	2	334	30–66	93–135	36.42	8.30
GhGRF12D	Gh_D13G1673	D13	50,330,408–50,334,209	996	2	331	27–63	90–132	36.13	8.29
GhGRF13A	Gh_A10G0492	A10	5,324,275–5,325,500	987	2	328	8–44	75–117	36.51	9.02
GhGRF14A	Gh_A13G1692	A13	75,869,496–75,870,119	546	1	181	12–48	75–117	20.73	9.26
GhGRF14D	Gh_D13G2042	D13	55,933,517–55,934,140	546	1	181	12–48	75–117	20.99	9.48
GhGRF15A	Gh_A11G0749	A11	7,286,137–7,288,429	1338	3	445	70–104	141–183/337–378	48.82	9.59
GhGRF15D	Gh_D11G0870	D11	7,509,881–7,512,169	1338	3	445	70–104	141–183/337–378	48.63	9.58
GhGRF16A	Gh_A08G1584	A08	94,278,338–94,283,477	774	3	257	65–101	159–189	27.79	8.89
GhGRF16D	Gh_D08G1891	D08	56,643,392–56,644,288	744	2	247	65–101	137–179	26.71	8.89
GhGRF17A	Gh_A12G2042	A12	83,346,587–83,347,484	729	2	242	58–94	130–172	26.37	9.10
GhGRF17D	Gh_D12G2219	D12	55,327,073–55,327,959	804	1	267	83–119	155–197	29.24	9.14

Note: *bp* Base pair, *Chr* Chromosome, *aa* Amino acid, *MW* Molecular weight, *kDa* Kilodalton, *pI* Isoelectric point

**Fig. 3:**
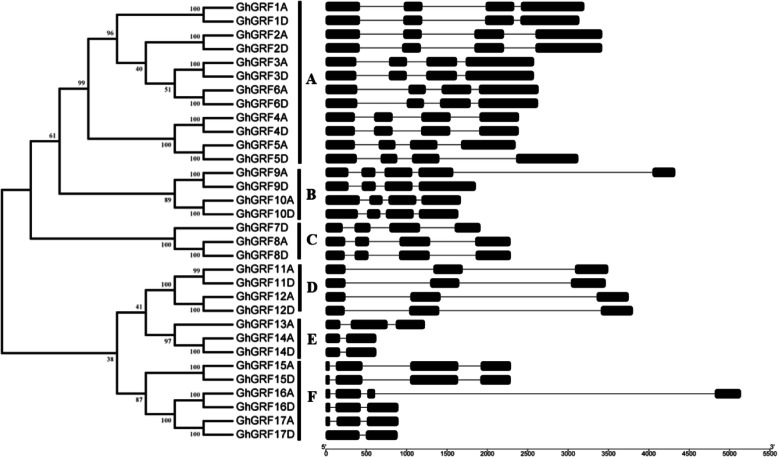
Phylogenetic tree and gene structure of GRF protein genes in *G. hirsutum*. Six phylogenetic clades were clustered. Exons and introns are represented by black boxes and lines, respectively. **a**-**f** represent six phylogenetic clades with different intron/exon structure

While all putative *GhGRF* genes contain predicted introns (Fig. 3), they also exhibit considerable variation, in both length and number. In general, homoeologous *GRF* genes show highly similar intron patterns (see exceptions below); however, intron structure among homoeologous pairs can exhibit variation in intron number (1 to 4) and length. Three of the homoeologous gene pairs did exhibit divergence in structure between homoeologs, namely *GhGRF9A* vs *GhGRF9D*, *GhGRF16A* vs *GhGRF16D* and *GhGRF17A* vs *GhGRF17D*. In two of the three cases (i.e., *GhGRF9*, *GhGRF16*), the acquisition of a splice site in the A-homoeolog led to an additional, large intron. For *GhGRF17A/D*, however, the D-homoeolog exhibits loss of splicing for the first intron, resulting in read-through transcription. Characterization of parental (in the diploids) gene structure for these three homoeologs suggests that this structural variation represents inherited, parental divergence. Phylogenetic analysis of the GRF gene family combined with intron/exon structure characterization naturally divides the family into six groups, here designated A-F, containing 3 to 12 genes (Fig. 3).

### Phylogenetic analysis of GRF proteins in *Gossypium*

**Fig. 4:**
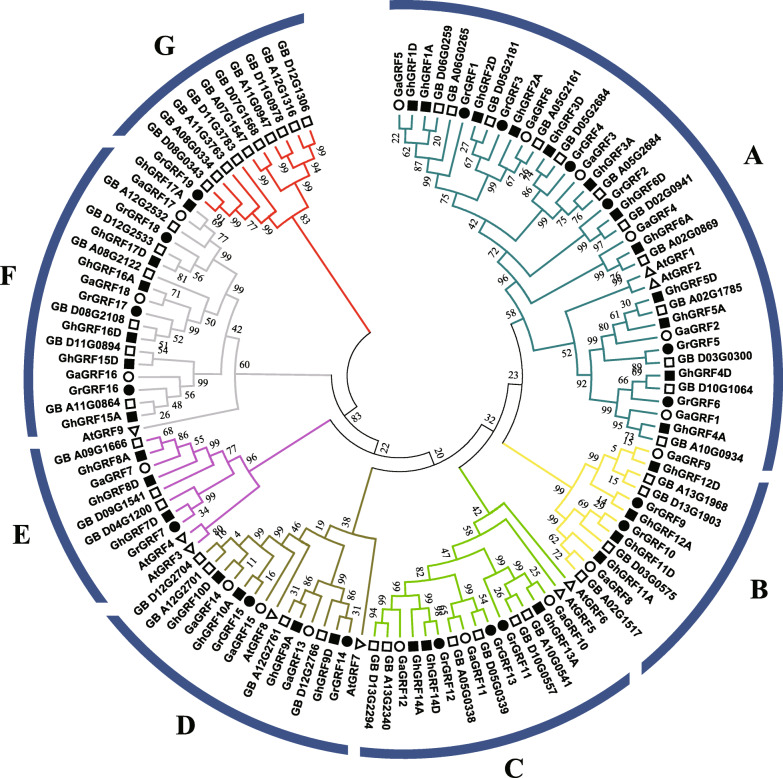
Phylogenetic analysis of GRF proteins from three *Gossypium* species and *Arabidopsis thaliana*. The phylogenetic tree was established with entire protein sequences with ML methods. The numbers on the branches indicate bootstrap support values from 1000 replications. The scale represents the units of the number of amino acid substitutions per site. The protein sequences used in the phylogenetic analysis are listed in Table 1. **a**-**g** represent seven phylogenetic clades

The general conservation of GRF genes between the two homoeologous genomes of *G. hirsutum* suggests minimal loss and/or gain since the divergence of the progenitor diploid genomes. We specifically assessed this using the protein sequences of 114 cotton GRFs (*G. hirsutum*, 32; *G. barbadense*, 45; *G. raimondii*, 19; and *G. arboreum*, 18; see methods) with 9 *Arabidopsis thaliana* GRFs for phylogenetic analysis (Fig. 4). The six previously designated clades (A–F) were again recovered plus extra clade “G”, with 1–2 *A. thaliana* genes associated with each clade except clades B and G, which are composed of *Gossypium* GRFs only.

Overall, the expected diploid-polyploid topology is reflected in the tree for each set of orthologous/homoeologous genes, indicating general preservation during diploid divergence and through polyploid evolution. That is, the number of GRF genes in *G. hirsutum* was generally additive with respect to the model diploid progenitors (*G. raimondii* and *G. arboreum*), with each homoeolog (A_t_ or D_t_) sister to their respective parental copies.

Although the GRF family exhibits general preservation, a few deviations were noted. For example, Clade C exhibits evidence of homoeolog loss; that is, the D copy of *GhGRF13* is missing from polyploid genome, as is both copy of *GaGRF11/GrGRF13* (one from each subgenome). In clade D, genes related to *AtGRF8* exhibit duplication in *G. arboreum* only, while the A-homoeolog from either polyploid species was not recovered from the genome sequence. This may indicate a duplication in *G. arboreum* after divergence from the polyploid ancestor coupled with loss in the A-subgenome of the polyploid. The evolution leading to clade E is less straightforward in that it is composed of eight genes, including one from each diploid progenitor and three from each tetraploid species. The phylogenetic topology, however, does not suggest a simple duplication of one homoeologous copy; rather, the homoeologous copies of *GhGRF8* are placed sister first to each other and then to *G. arboreum* only. The remaining GRF from *G. hirsutum*, *GhGRF7D*, is sister to the only *G. raimondii* GRF of this clade and has no inferred homoeologous A_T_ copy. Given this pattern of relationships and that *G. raimondii* has one additional GRF (relative to *G. arboreum*; see next section), it seems possible that *GhGRF7D* represents a uniquely inherited GRF present in the D ancestor only and that the *G. raimondii* ortholog of *GhGRF8D* was lost after polyploidization. Alternatively, this pattern could reflect a non-syntenic duplication of *GhGRF8D*, followed by conversion of the original *GhGRF8D* into an A-like copy. It bears noting, however, that these observed deviations could be due to errors in the genome sequences; however, the general preservation of GRF copies in the expected relationships suggests general robustness of the analysis. Clade G contains 11 GRF genes, one from *G. raimondii* and the other ten from *G. barbadense*, also indicative of substantial duplication in *G. barbadense*. These new genes belongs to one family and might specially originate in *G. raimondii* and the tetraploid progenitor of *G. barbadense* or undergone the homoeolog loss in *G. arboreum* and *G. hirsutum*.

### Dynamic evolution of *GRF* family genes in plants

**Fig. 5:**
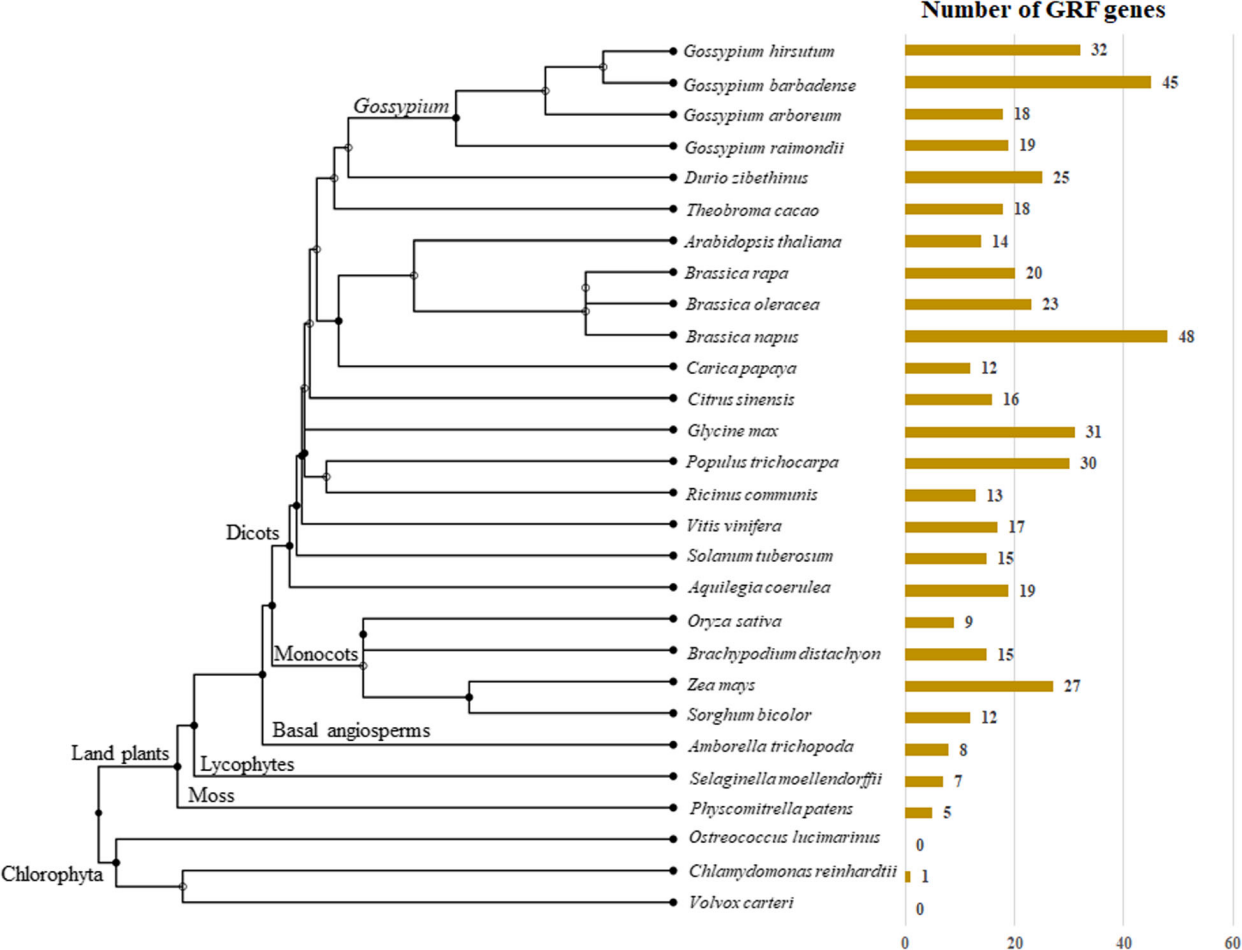
Phylogenetic distribution of the GRF transcription factor family genes in Plantae. The phylogenetic tree of 28 plant species are constructed by the TIMETREE (http://www.timetree.org/). The number of GRF family genes identified are presented on the right

We further evaluated the general preservation of GRF genes in plants using 28 representative species and the same search criteria (see methods and Fig. 5). In Chlorophyta, only one *GRF* gene was identified as putatively encoding a GRF protein (in *Chlamydomonas reinhardtii* only); however, all land plant species surveyed recovered a minimum of five putative *GRF* genes in *Physcomitrella patens* and nearly double were found in angiosperms (8 in the basal angiosperm, *Amborella trichopoda*). Among angiosperms, GRF copy number varied from the minimum of 8, in *Amborella trichopoda*, to 48 copies in the tetraploid *Brassica napus.* Four monocot species were included, where between 9 (in rice) to 27 (in maize) copies were detected. Notably, this high copy number in maize is slightly more than double the copy number in sorghum, likely reflective of the duplicated history of maize. GRF copy numbers in the eudicot species surveyed varied even more, from 12 putative *GRF* genes in *Carica papaya* to 48 in *Brassica napus*, notably another polyploid species genome.

Comparatively, *Gossypium* GRF copy number is generally stable. We recovered 19 and 18 GRF genes from *G. raimondii* (model D-genome ancestor) and *G. arboreum* (model A-genome ancestor), respectively, dispersed among 10 of the 13 chromosomes (Additional file 4: Figure S2). The allotetraploid cotton species included here both contain a roughly additive number of genes; however, while *G. hirsutum* appears to have lost genes (34 GRF genes, versus the additive expectation of 36), *G. barbadense* has gained 9 additional predicted copies through duplication (45 versus 36; Fig. 5).

### Expression changes of *GhGRF* genes in *G. arboreum*, *G. hirsutum,* and *G. barbadense* under salt stress

GRF genes have been linked to various aspects of growth, development, and response to stress. Long-term effects of salt stress affect all aspects of the plant life cycle, from seed germination through growth and development to future reproductive potential [36]. While the effects of salt stress on roots is often considered [37, 38], equally important are the consequent physiological changes in leaves where the plants exclude sodium ions, potassium/potassium balance, and reduce water loss [39–45]. Previously, a single cotton GRF gene (*GhGRF1* [27];) was associated with responses to salt stress; however, the contributions of the remaining family members to salt stress responses is unknown.

**Fig. 6:**
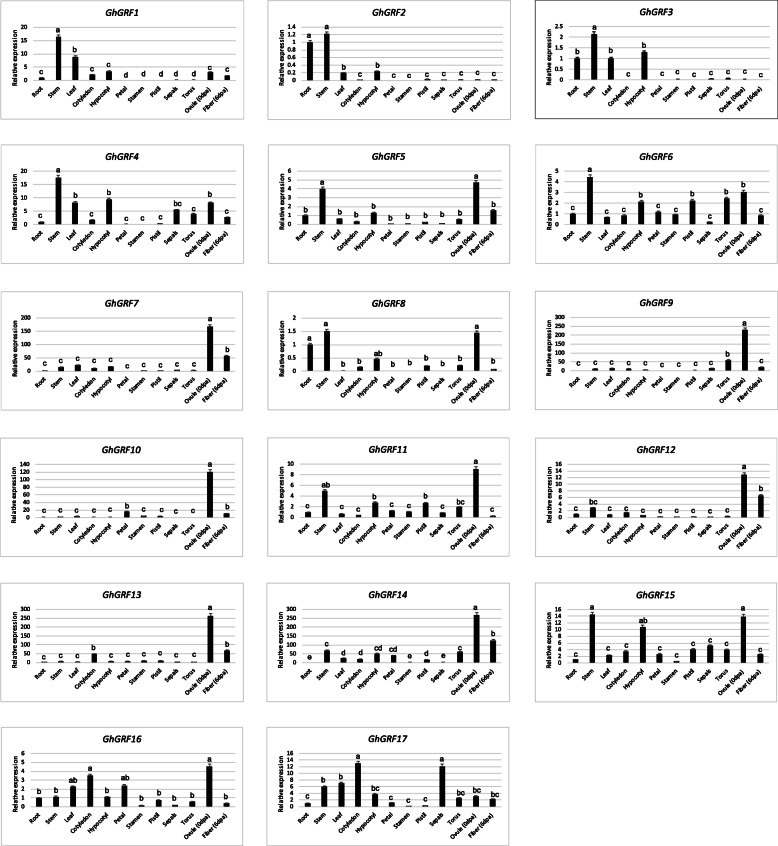
Expression of *GhGRF* genes in different tissues analyzed by real-time quantitative reverse transcription polymerase chain reaction (qRT-PCR). Twelve tissues, including roots, stems, leaves, cotyledon, hypocotyl petal, stamen, pistil, sepals, torus, ovules (0dpa) and fiber (6dpa), were collected from the continuing growing cotton plants. Relative gene expression levels are normalized to *histone-3* gene (*GhHIS3*) values. Error bars indicate SD (*n* = 3). Statistically significant differences (“a” is different from “b” or “c”, α = 0.01 level) of expression values are indicated with different letters with analysis of variance in R (https://www.r-project.org/)

To evaluate the contribution of these *GhGRF* genes to leaf physiology, we first evaluated their expression under normal conditions in 12 different cotton tissues, including root, stem, leaf, cotyledon, hypocotyl, petal, stamen, pistil, sepals, receptacle, ovules (0 dpa) and fiber (6 dpa), using both qPCR and existing RNA-seq data; homoeologs were not distinguished in the qPCR analysis. Most *GhGRF* genes exhibited different expression patterns among the various tissues; however, certain generalities were observed. Several tissues exhibited distinct paralog preference in our qPCR survey. For example, six of the examined *GhGRF* genes (*GhGRF5*, *6*, *11*, *15*–*17*; Fig. 6) were broadly expressed across tissues, whereas six other *GhGRF* genes (*GhGRF7*, *9*, *10*, *12*–*14*; Fig. 6) were mainly expressed in the ovule and fiber samples, with limited expression in most other tissues. Most *GhGRF* genes, including *GhGRF5*-*GhGRF12*, were expressed in the developing ovule (Fig. 6), which transitioned to preferential usage of seven genes (*GhGRF1*, *GhGRF4*, *GhGRF7*, *GhGRF10*, *GhGRF13* and *GhGRF14*) during the primary elongation stage of cotton fiber development (6 dpa). Transcripts of *GhGRF1*, *GhGRF2*, *GhGRF3* and *GhGRF4* were most abundant in stem and root, whereas *GhGRF16* and *GhGRF17* were preferentially expressed in cotyledon. Over half of the *GhGRF* genes showed the relatively high level of transcripts in leaves.

RNA-sequencing (RNA-Seq) data (Additional file 5: Figure S3) obtained from the *G. hirsutum* genome sequencing project [30] and the ccNET database [46] are generally congruent with the expression patterns revealed by qPCR (Fig. 6), although there exist a few discrepancies (Additional file 5: Figure S3). These are generally limited to relative abundance among tissues, such as *GhGRF14*, which exhibited the highest expression in pistil by the RNA-Seq data, but showed higher expression in ovule (0 dpa) and fiber (6 dpa) using qPCR (Fig. 6). Expression in the leaf RNA-seq was much lower for most genes, except *GhGRF1*, and the observation may be attributable to differences in leaf maturity. Generally, however, the two expression analyses suggest a complex pattern of paralog usage among GhGRF genes indicative of multiple biological functions during plant growth and development.

**Fig. 7:**
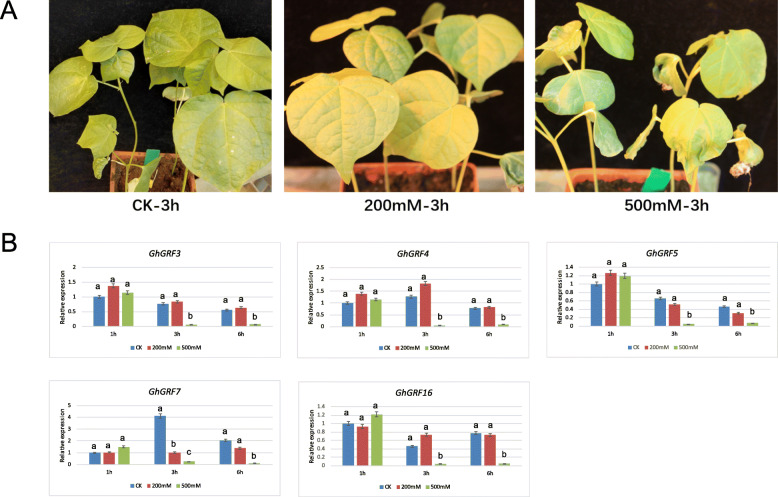
Phenotypes and expression profile analyses of *GhGRF* genes under different concentrations of salt stress conditions analyzed by qRT-PCR. **a:** Phenotypes of upland cotton *G. hirsutum* cv. R15 under different concentrations of NaCl treatment. **b:** Relative gene expression levels in leaves after 1, 3, and 6 h of the treatment with 0, 200, and 500 mM NaCl. CK, 0 mM. The *G. hirsutum histone-3* (*GhHIS3*) and *GhUBQ7* genes were used as the internal reference. Error bars indicate SD (*n* = 3). Statistically significant differences (α = 0.01 level) of expression values are indicated with different letters with analysis of variance in R (https://www.r-project.org/)

**Fig. 8:**
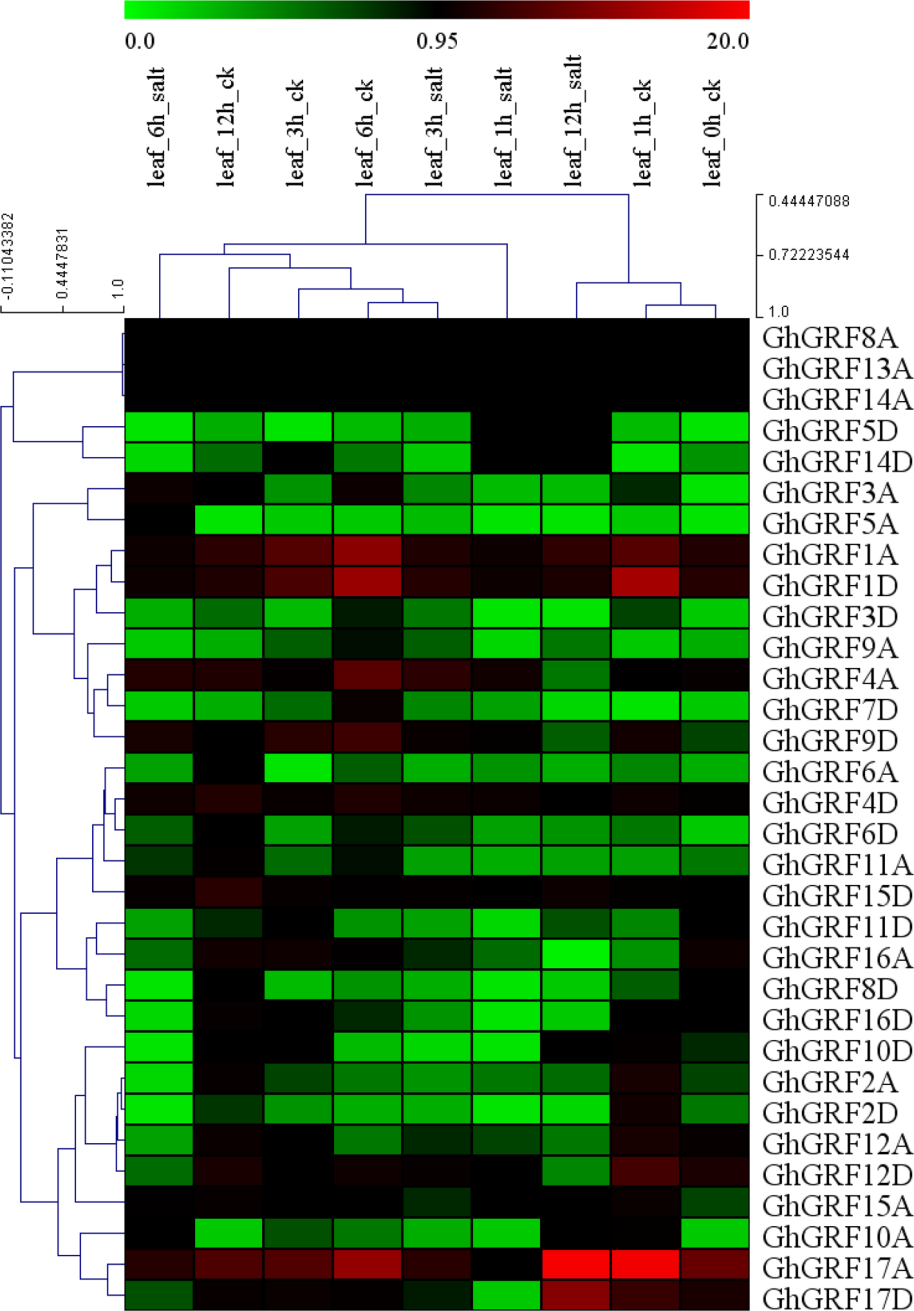
Expression patterns of *GhGRF* genes in response to salt stresses from RNA-seq data. The RNA-seq data were downloaded from Zhang et al., 2015 and re-analyzed the RPKM values of five time points (0, 1, 3, 6 and 12 h) after salt treatments

Upland cotton can experience stress [38] due to salt accumulation in the soil. Since *GRF*s have been shown to respond to salt stress responses in other plants [47], we observed phenotypic changes under two concentrations of NaCl and concurrently analyzed *GhGRF* expression profiles using qRT-PCR (Fig. 7). Phenotypic changes in the leaves of Upland cotton were observed using both 200 and 500 mM NaCl over a period of 6 h. With 200 mM NaCl, leaves of *G. hirsutum* experienced yellowing only (indicative of chloroplast degradation), whereas treatment with 500 mM NaCl resulted in leaf yellowing and withering (Fig. 7a). Expression changes (relative to the control; Fig. 7b) were not significant for the 200 mM NaCl at three time-points (1 h, 3 h and 6 h) post-treatment; however, the higher NaCl concentration (500 mM) resulted in down-regulation of five genes (*GhGRF3*, *GhGRF4*, *GhGRF5*, *GhGRF7*, and *GhGRF16*; Fig. 7b). No *GhGRF* genes experienced up-regulation under salt stress, which is congruent with the observation that salt stress leads to diminished growth. The RNA-seq data derived from the *G. hirsutum* genome and ccNET [46] generally concur with these results. For example, 3 of the 5 genes downregulated here (i.e., *GhGRF3*, *GhGRF4*, and *GhGRF16*) also exhibited significantly decreased expression levels (≥2-fold change) at four time-points during NaCl stress in the available RNA-seq data (Fig. 8). These three GRFs in particular may be commonly responsive to salt stress and warrant further functional characterization.

**Fig. 9:**
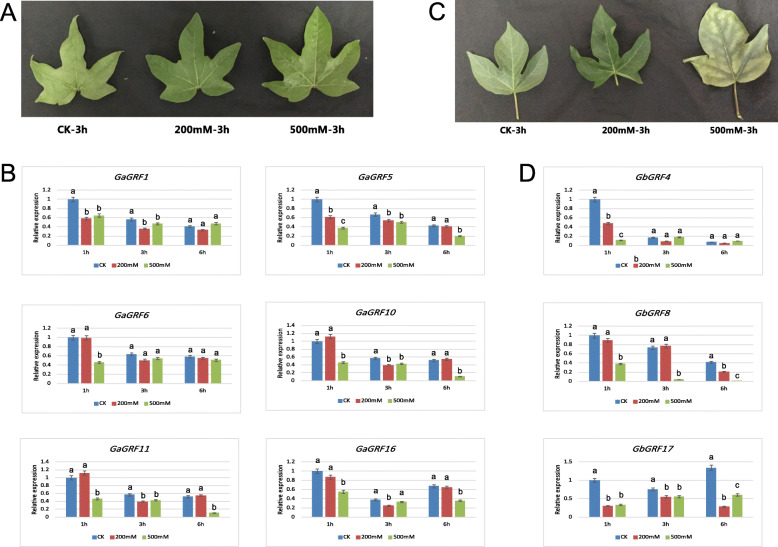
Phenotypes and expression profile analyses of *GaGRF* and *GbGRF* genes under different concentrations of salt stress conditions analyzed by qRT-PCR in *G. arboreum* and *G. barbadense*, respectively. **a:** Phenotypes of diploid cotton *G. arboreum* cv. Shiyixia1 under different concentrations of NaCl treatment. **b:** Relative gene expression levels in leaves after 1, 3, and 6 h of the treatment with 0, 200, and 500 mM NaCl. CK, 0 mM. **c:** Phenotypes of *G. barbadense* cv. H7124 under different concentrations of NaCl treatment. **d:** Relative gene expression levels in leaves after 1, 3, and 6 h of the treatment with 0, 200, and 500 mM NaCl. CK, 0 mM. The *G. arboreum* and *G. barbadense histone-3* (*HIS3*) and *UBQ7* genes were used as the internal reference. Error bars indicate SD (*n* = 3). Statistically significant differences (α = 0.01 level) of expression values are indicated with different letters with analysis of variance in R (https://www.r-project.org/)

We also observed the phenotypic changes of *G. arboreum* and *G. barbadense* under two concentrations of NaCl and concurrently analyzed the *GaGRF* and *GbGRF* expression profiles by qRT-PCR (Fig. 9). Phenotypic changes in the leaves of *G. arboreum* and *G. barbadense* were observed using both 200 and 500 mM NaCl over a period of 6 h. Although neither 200 nor 500 mM NaCl treatment resulting in yellowing or withering of *G. arboreum* leaves (Fig. 9a), downregulation of six *GaGRF* genes (Fig. 9b) was nevertheless detected at three time-points (1 h, 3 h and 6 h) post-treatment. Notably, only two of these genes, i.e., *GaGRF5* and *GaGRF16*, were also down-regulated in *G. hirsutum.* Similar to *G. hirsutum*, *G. barbadense* was modestly affected by low salt treatment, with leaf withering beginning at 200 mM NaCl and both leaf yellowing and withering present at 500 mM NaCl (Fig. 9c); however, here only three *GbGRF* genes (Fig. 9d) exhibited down-regulation, only one of which (*GbGRF4*) exhibited downregulation in either *G. hirsutum* or *G. arboreum*. Of the three genes downregulated in *G. barbadense*, expression changes of *GbGRF17* was significant for the 200 mM and 500 mM NaCl at three time-points (1 h, 3 h and 6 h) post-treatment. These results demonstrate that (1) *G. arboreum* has better salt tolerance than both *G. hirsutum* and *G. barbadense*, congruent with the previous observations [28, 48, 49], and (2) GRF responses to salt stress overlap, but are not consistent, among species.

## Discussion

The GRF transcription factor family of genes has been investigated in a number of plant species, including *Arabidopsis thaliana* [20, 50–53], *Zea mays* [35], *Brassica napus* [54], *Brassica rapa* [55], *Oryza sativa* [56–58], *Brachypodium distachyon* [59] and *Solanum lycopersicum* [22]. The family generally contains conserved functional regions, including a protein-protein interaction QLQ (glutamine, leucine, glutamine) domain that was involved in chromatin remodeling [60] and a plant-specific WRC (tryptophan, arginine, cysteine) motif relevant to DNA binding and targeting of the TF to the nucleus [14, 58]. The conservation of these domains among GRF genes facilitates computational identification of the family from new and emerging genomes. Here, we identify 32 GRF genes from the allotetraploid *G. hirsutum*, and show that these genes are generally conserved among the diploid progenitors and the cultivated allotetraploid cottons. Analysis of the GRF genes in an additional cotton species, as well as representatives of diverse plant lineages, show that the family is somewhat labile but the most remarkable changes in copy number are attributable to retention after genome duplication. In tetraploid cottons, the gene copy number is nearly additive in *G. hirsutum,* but contains 25% more paralogs in the related (and also domesticated) *G. barbadense* genome. Phylogenetic analysis indicated that these additive genes belongs to one family and specially originate in *G. barbadense*. Further research is required to discern what functional relevance these additional copies have.

## Conclusions

We systematically characterized cotton GRF family genes using bioinformatic and phylogenetic approaches and gene expression analyses. We analyzed gene structures, chromosomal locations, intron-exon organizations, phylogenetic relationships and expression profile patterns in different cotton tissues and under salt stress condition to predict their possible biological functions. *GhGRF* genes are variably expressed in different cotton tissues with particularly high expression in ovules. The decreased expression of several *GhGRF* genes in response to salt stress treatments implies their function in the regulations of growth and development under the abiotic stress conditions. Together, our results provide data to facilitate the functional identification of the GRF genes in cotton plant growth, development and stress tolerance.

## Methods

### Identification of GRF family genes and GRF proteins in diploid and tetraploid *Gossypium* species

We downloaded the genome sequences of four cotton species from the CottonGen database [61], including *G. raimondii* [25], *G. arboreum* [27], *G. hirsutum* [30], and *G. barbadense* [62]. To identify all putative GRF transcription factor proteins in each genome assembly, the GRF protein conserved domains (PF08879 for WRC domain and PF08880 for QLQ domain) were used to develop a Hidden Markov Model [63] profile matrix via the hmmbuild program from the HMMER package [64] using default parameters. This HMM profile matrix was used in conjunction with hmmersearch with default parameters against three *Gossypium* genome databases, i.e., *G. raimondii, G. arboreum,* and *G. hirsutum*, to identify putative GRF genes (*GhGRF*s) that contain the relevant conserved protein domains. Genes were considered candidate GRFs if they harbored WRC and QLQ domains within the N-terminal [13]. Previously identified *GRF* gene sequences from *Arabidopsis thaliana* (*AtGRF*s) were retrieved from the TAIR database [64] for phylogenetic comparison. The presence of conserved domains in each *Arabidopsis* gene was verified using the SMART conserved domain search tool [65] and Pfam databases [66]. The same method was used to identify the number of GRF genes in other plant genera/species (Additional file 1: Table S1).

### Chromosomal location and gene structure analyses

Chromosomal locations for each of the above identified *GhGRFs* were extracted from the genome annotation gff3 file [30]. Chromosomal locations of the predicted *GhGRF*s was visualized using MapChart [67], and the exon-intron structure of each gene was displayed using the online tool GSDS 2.0 [68]. The number of amino acids, molecular weight (MW), and theoretical isoelectric point (pI) of putative *GhGRF* proteins were determined using the ProtParam tool [69].

### Sequence alignment and phylogenetic analyses

Complete protein-coding sequences for each of these genes from all three cotton species and *Arabidopsis* were aligned using MAFFT with the G-INS-i algorithm [70]. Phylogenetic analyses based on the whole protein sequences were performed using Neighbor-Joining (NJ) and Maximum Likelihood (ML). The NJ tree was constructed using MEGA version 5.03 [71] by sampling 1000 bootstrap replicates. The ML tree was also built using MEGA version 5.03, using the general time reversible (GTR) model, including rate variation among sites (+G) and invariable sites (+I; full model = GTR + G + I), and running 1000 bootstrap replicates of the data.

### Transcriptome data-based gene expression analyses

Raw RNA-Seq data for *G. hirsutum* seed, root, stem, leaf, torus, petal, stamen, ovary, calyx, ovule (− 3 dpa, − 1 dpa, 0 dpa, 1 dpa, 3 dpa, 5 dpa, 10 dpa, 20 dpa, 25dpa, 35dpa) and fiber (5 dpa, 10 dpa, 20 dpa, 25dpa) were downloaded from the NCBI Sequence Read Archive (https://www.ncbi.nlm.nih.gov/bioproject/PRJNA248163) [30], represented by one library each. Reads were mapped to *G. hirsutum* genome [30] via HISAT2 software with default parameters, and read abundance with calculated via StringTie [72, 73]. Read counts were normalized in R3.2 using RUVSeq [74] and the internal control reference gene *GhUBQ7* (accession number: DQ116441), which is detected at relatively constant levels across different cotton samples [75]. Potential batch-effects were corrected by an improved version of ComBat, ComBat-seq [76]. Gene expression was estimated by Ballgown [86], using fragments per kilobase million (FPKM) values to calculate the gene expression levels across libraries. Expression levels of *G. hirsutum* leaf RNA-Seq data (in FPKM) for each *GhGRF* gene under salt stress (time points: 0, 1, 3, 6, 12 h) were retrieved from the ccNET database [46]. Genes were considered differentially expressed if expression varied more than two-fold change with a *p*-value of less than 0.05. Multiple Experiment Viewer (MeV) [77] was used to display the gene expression patterns from the reported FPKM values.

### Plant cultivation and treatment

To generate new expression information via qRT-PCR, we grew representatives of *G. hirsutum, G. arboreum,* and *G. barbadense*. For *G. hirsutum,* seeds of *G. hirsutum* cv. R15 [78], were germinated in potting soil in a growth chamber, and the resulting seedlings were maintained in a controlled environment at 28 °C day/20 °C night, with a 16-h light/8-h dark photoperiod. Roots, stems, leaves, cotyledons, and hypocotyls were collected from the three-week old plants, and additional samples were collected from older, flowering plants; these include petal, stamen, pistil, sepals, torus, ovules (0 dpa (days post anthesis)) and fiber (6 dpa). Three biological replicates were collected for each sample, each with three technical replicates. For salt treatment, 28-day old plants were sprayed with 200 and 500 mM NaCl solution after surfactant (Triton X-100) treatments. Leaves from salt-treated plants were collected at 0 (control), 1, 3, and 6 h post-NaCl treatment for further expression analyses. All plant tissues were frozen in liquid nitrogen immediately after collection and stored at − 80 °C until RNA extraction. All treatments was sampled at least three times.

Similarly, *G. arboreum* cv. Shixiya1 and *G. barbadense* H7124 were grown for qRT-PCR of salt-exposed leaf tissue timepoints only. For this experiment, seeds of *G. arboreum* cv. Shixiya1 were provided by Prof. Tianzhen Zhang and *G. barbadense* H7124 seeds were provided from the Esquel Group. These two *Gossypium* species were planted in the Damao field in Sanya, Hainan Province, China. For salt treatment, 50-day old plants were sprayed with 200 and 500 mM NaCl solution after surfactant (Triton X-100) treatment. Leaves from salt-treated plants were collected at 0 (control), 1, 3, and 6 h post-NaCl treatment as above.

### RNA extraction, cDNA synthesis and qRT-PCR expression analyses

Total RNAs from cotton tissues were extracted using the RNAprep pure plant kit (TIANGEN, Shanghai, China) according to the manufacturer’s protocol. The resulting RNAs were treated with DNase I prior to synthesizing cDNA with oligo (dT) primers and M-MLV Reverse Transcriptase (Invitrogen); these products were diluted 5-fold before use. For quantitative real-time PCR (qRT-PCR), Primer5 software was used to design gene-specific forward and reverse primers (Additional file 2: Table S2). As these primers are not homoeolog specific, both copies were amplified when retained in duplicate. Analyses were performed with SYBR-Green PCR Mastermix (TaKaRa) on a cycler (Mastercycler RealPlex; Eppendorf Ltd., Shanghai, China). The *G. hirsutum histone-3* (*GhHIS3*, AF024716) and *GhUBQ7* (accession number: DQ116441) genes were used as internal references, and the relative amount of amplified product was calculated following the 2-∆∆Ct method [79]. For the *G. hirsutum* samples, relative expression levels among different organs were normalized by calibrating with the root sample from that plant. The root sample was washed with DEPC sterile water three times before extracting the RNA.

## Supplementary information

**Additional file 1: Table S1.** 28 plant species used for identifying the number of GRF family genes.

**Additional file 2: Table S2.** List of forward and reverse primers used for qRT-PCR analyses

**Additional file 3: Figure S1.** Multiple alignment of *GhGRF* (*Gossypium hirsutum* GRF) protein sequences

**Additional file 4: Figure S2.** Chromosome distribution of *GaGRF* (*Gossypium arboreum* GRF) and *GrGRF* (*Gossypium raimondii* GRF) genes.

**Additional file 5: Figure S3.** Expression patterns of *GRF* genes in *G. hirsutum* using RNA-seq data. The RNA-seq data expression profiles were from Zhang et al., (2015) and ccNET database [46]. FPKM represents fragments per kilobase of exon model per million mapped reads. DPA, days post anthesis

## Data Availability

The genome sequences of four cotton species and the genome annotation gff3 file were downloaded from the CottonGen database (https://www.cottongen.org/data/download) [61]. GRF gene sequences from *Arabidopsis thaliana* were retrieved from the TAIR database (https://www.arabidopsis.org/servlets/Search?type=general&search_action=detail&method=1&show_obsolete=F&name=GRF&sub_type=gene&SEARCH_EXACT=4&SEARCH_CONTAINS=1) [65]. Raw RNA-Seq data for *G. hirsutum* seed, root, stem, leaf, torus, petal, stamen, ovary, calyx, ovule and fiber were downloaded from the NCBI Sequence Read Archive (https://www.ncbi.nlm.nih.gov/bioproject/PRJNA248163) (NCBI Sequence Read Archive SRR1695173, SRR1695174, SRR1695175, SRR1695177, SRR1695178, SRR1695179, SRR1695181, SRR1695182, SRR1695183, SRR1695184, SRR1695185, SRR1695191, SRR1695192, SRR1695193,SRR1695194, SRR1768504, SRR1768505, SRR1768506, SRR1768507, SRR1768508, SRR1768509, SRR1768510, SRR1768511, SRR1768512, SRR1768513, SRR1768514, SRR1768515, SRR1768516, SRR1768517, SRR1768518 and SRR1768519) [30]. The *G. hirsutum histone-3* (*GhHIS3*, AF024716) and *GhUBQ7* (accession number: DQ116441) genes were downloaded from the National Center for Biotechnology Information (NCBI) database, which were used as internal references. All other data generated or analyzed during this study are included in this published article and its Additional files.

## Abbreviations

GRFs: Growth regulating factors
DPA: Days post anthesis
FPKM: Fragments per kilobase of transcript per million mapped fragments
*G. arboreum*: *Gossypium arboreum*
*G. hirsutum*: *Gossypium hirsutum*
*G. raimondii*: *Gossypium raimondii*
MW: Molecular weight
pI: Isoelectric point
qRT-PCR: Quantitative real-time polymerase chain reaction
