# Supplementary material for: Genome-wide characterization of the GRF family and their roles in response to salt stress in Gossypium
Source: BMC Genomics. 2020 Aug 24;21:575. doi: 10.1186/s12864-020-06986-0 (PMC7444260; doi:10.1186/s12864-020-06986-0)
Supplement: Supplementary file 1 — Additional file 1: Table S1. 28 plant species used for identifying the number of GRF family genes. [file 12864_2020_6986_MOESM1_ESM.docx]

**Table S1.** 28 plant species used for identifying the number of GRF family genes.

| Organisms | Citations |
| --- | --- |
| *Gossypium hirsutum* | Hu et al., 2019^[1]^ |
| *Gossypium barbadense* | Hu et al., 2019^[1]^ |
| *Gossypium arboreum* | Li et al., 2014^[2]^ |
| *Gossypium raimondii* | Paterson et al., 2012^[3]^ |
| *Durio zibethinus* | Teh et al., 2017^[4]^ |
| *Theobroma cacao* | Argout et al., 2011^[5]^ |
| *Arabidopsis thaliana* | Arabidopsis Genome I et al., 2006^[6]^ |
| *Brassica rapa* | Wang et al., 2012^[7]^ |
| *Brassica oleracea* | Liu et al., 2014^[8]^ |
| *Brassica napus* | Chalhoub et al., 2014^[9]^ |
| *Carica papaya* | Ming et al., 2008^[10]^ |
| *Citrus sinensis* | Xu et al., 2013^[11]^ |
| *Glycine max* | Schmutz et al., 2010^[12]^ |
| *Populus trichocarpa* | Tuskan et al., 2006^[13]^ |
| *Ricinus communis* | Chan et al., 2010^[14]^ |
| *Vitis vinifera* | Jaillon et al., 2007^[15]^ |
| *Solanum tuberosum* | Xu et al., 2011^[16]^ |
| *Aquilegia coerulea* | Filiault et al., 2018^[17]^ |
| *Oryza sativa* | Yu et al., 2002^[18]^ |
| *Brachypodium_distachyon* | International Brachypodium I et al., 2010^[19]^ |
| *Zea mays* | Schnable et al., 2009^[20]^ |
| *Sorghum bicolor* | Paterson et al., 2009^[21]^ |
| *Amborella trichopoda* | Amborella Genome P et al., 2013^[22]^ |
| *Selaginella moellendorffii* | Banks et al., 2011^[23]^ |
| *Physcomitrella patens* | Rensing et al., 2008^[24]^ |
| *Ostreococcus lucimarinus* | Palenik et al., 2007^[25]^ |
| *Chlamydomonas reinhardtii* | Blaby et al., 2014^[26]^ |
| *Volvox carteri* | Prochnik et al., 2010^[27]^ |

1. Hu Y, Chen J, Fang L, Zhang Z, Ma W, Niu Y, Ju L, Deng J, Zhao T, Lian J *et al*: *Gossypium barbadense* and *Gossypium hirsutum* genomes provide insights into the origin and evolution of allotetraploid cotton. *Nature genetics* 2019.

2. Li F, Fan G, Wang K, Sun F, Yuan Y, Song G, Li Q, Ma Z, Lu C, Zou C *et al*: Genome sequence of the cultivated cotton *Gossypium arboreum*. *Nature genetics* 2014, 46(6):567-572.

3. Paterson AH, Wendel JF, Gundlach H, Guo H, Jenkins J, Jin D, Llewellyn D, Showmaker KC, Shu S, Udall J *et al*: Repeated polyploidization of *Gossypium* genomes and the evolution of spinnable cotton fibres. *Nature* 2012, 492(7429):423-427.

4. Teh BT, Lim K, Yong CH, Ng CCY, Rao SR, Rajasegaran V, Lim WK, Ong CK, Chan K, Cheng VKY *et al*: The draft genome of tropical fruit durian (*Durio zibethinus*). *Nature genetics* 2017, 49(11):1633-1641.

5. Argout X, Salse J, Aury JM, Guiltinan MJ, Droc G, Gouzy J, Allegre M, Chaparro C, Legavre T, Maximova SN *et al*: The genome of *Theobroma cacao*. *Nature genetics* 2011, 43(2):101-108.

6. Arabidopsis Genome I: Analysis of the genome sequence of the flowering plant *Arabidopsis thaliana*. *Nature* 2000, 408(6814):796-815.

7. Wang X, Wang H, Wang J, Sun R, Wu J, Liu S, Bai Y, Mun JH, Bancroft I, Cheng F *et al*: The genome of the mesopolyploid crop species *Brassica rapa*. *Nature genetics* 2011, 43(10):1035-1039.

8. Liu S, Liu Y, Yang X, Tong C, Edwards D, Parkin IA, Zhao M, Ma J, Yu J, Huang S *et al*: The *Brassica oleracea* genome reveals the asymmetrical evolution of polyploid genomes. *Nat Commun* 2014, 5:3930.

9. Chalhoub B, Denoeud F, Liu S, Parkin IA, Tang H, Wang X, Chiquet J, Belcram H, Tong C, Samans B *et al*: Plant genetics. Early allopolyploid evolution in the post-Neolithic *Brassica napus* oilseed genome. *Science* 2014, 345(6199):950-953.

10. Ming R, Hou S, Feng Y, Yu Q, Dionne-Laporte A, Saw JH, Senin P, Wang W, Ly BV, Lewis KL *et al*: The draft genome of the transgenic tropical fruit tree papaya (*Carica papaya* Linnaeus). *Nature* 2008, 452(7190):991-996.

11. Xu Q, Chen LL, Ruan X, Chen D, Zhu A, Chen C, Bertrand D, Jiao WB, Hao BH, Lyon MP *et al*: The draft genome of sweet orange (*Citrus sinensis*). *Nature genetics* 2013, 45(1):59-66.

12. Schmutz J, Cannon SB, Schlueter J, Ma J, Mitros T, Nelson W, Hyten DL, Song Q, Thelen JJ, Cheng J *et al*: Genome sequence of the palaeopolyploid soybean. *Nature* 2010, 463(7278):178-183.

13. Tuskan GA, Difazio S, Jansson S, Bohlmann J, Grigoriev I, Hellsten U, Putnam N, Ralph S, Rombauts S, Salamov A *et al*: The genome of black cottonwood, *Populus trichocarpa* (Torr. & Gray). *Science* 2006, 313(5793):1596-1604.

14. Chan AP, Crabtree J, Zhao Q, Lorenzi H, Orvis J, Puiu D, Melake-Berhan A, Jones KM, Redman J, Chen G *et al*: Draft genome sequence of the oilseed species *Ricinus communis*. *Nature biotechnology* 2010, 28(9):951-956.

15. Jaillon O, Aury JM, Noel B, Policriti A, Clepet C, Casagrande A, Choisne N, Aubourg S, Vitulo N, Jubin C *et al*: The grapevine genome sequence suggests ancestral hexaploidization in major angiosperm phyla. *Nature* 2007, 449(7161):463-467.

16. Potato Genome Sequencing C, Xu X, Pan S, Cheng S, Zhang B, Mu D, Ni P, Zhang G, Yang S, Li R *et al*: Genome sequence and analysis of the tuber crop potato. *Nature* 2011, 475(7355):189-195.

17. Filiault DL, Ballerini ES, Mandakova T, Akoz G, Derieg NJ, Schmutz J, Jenkins J, Grimwood J, Shu S, Hayes RD *et al*: The Aquilegia genome provides insight into adaptive radiation and reveals an extraordinarily polymorphic chromosome with a unique history. *Elife* 2018, 7.

18. Yu J, Hu S, Wang J, Wong GK, Li S, Liu B, Deng Y, Dai L, Zhou Y, Zhang X *et al*: A draft sequence of the rice genome (*Oryza sativa* L. ssp. indica). *Science* 2002, 296(5565):79-92.

19. International Brachypodium I: Genome sequencing and analysis of the model grass *Brachypodium distachyon*. *Nature* 2010, 463(7282):763-768.

20. Schnable PS, Ware D, Fulton RS, Stein JC, Wei F, Pasternak S, Liang C, Zhang J, Fulton L, Graves TA *et al*: The B73 maize genome: complexity, diversity, and dynamics. *Science* 2009, 326(5956):1112-1115.

21. Paterson AH, Bowers JE, Bruggmann R, Dubchak I, Grimwood J, Gundlach H, Haberer G, Hellsten U, Mitros T, Poliakov A *et al*: The *Sorghum bicolor* genome and the diversification of grasses. *Nature* 2009, 457(7229):551-556.

22. Amborella Genome P: The Amborella genome and the evolution of flowering plants. *Science* 2013, 342(6165):1241089.

23. Banks JA, Nishiyama T, Hasebe M, Bowman JL, Gribskov M, dePamphilis C, Albert VA, Aono N, Aoyama T, Ambrose BA *et al*: The Selaginella genome identifies genetic changes associated with the evolution of vascular plants. *Science* 2011, 332(6032):960-963.

24. Rensing SA, Lang D, Zimmer AD, Terry A, Salamov A, Shapiro H, Nishiyama T, Perroud PF, Lindquist EA, Kamisugi Y *et al*: The Physcomitrella genome reveals evolutionary insights into the conquest of land by plants. *Science* 2008, 319(5859):64-69.

25. Palenik B, Grimwood J, Aerts A, Rouze P, Salamov A, Putnam N, Dupont C, Jorgensen R, Derelle E, Rombauts S *et al*: The tiny eukaryote Ostreococcus provides genomic insights into the paradox of plankton speciation. *Proceedings of the National Academy of Sciences of the United States of America* 2007, 104(18):7705-7710.

26. Blaby IK, Blaby-Haas CE, Tourasse N, Hom EFY, Lopez D, Aksoy M, Grossman A, Umen J, Dutcher S, Porter M *et al*: The Chlamydomonas genome project: a decade on. *Trends in plant science* 2014, 19(10):672-680.

27. Prochnik SE, Umen J, Nedelcu AM, Hallmann A, Miller SM, Nishii I, Ferris P, Kuo A, Mitros T, Fritz-Laylin LK *et al*: Genomic analysis of organismal complexity in the multicellular green alga *Volvox carteri*. *Science* 2010, 329(5988):223-226.
