# Supplementary material for: Genome-wide characterization of the GRF family and their roles in response to salt stress in Gossypium
Source: BMC Genomics. 2020 Aug 24;21:575. doi: 10.1186/s12864-020-06986-0 (PMC7444260; doi:10.1186/s12864-020-06986-0)
Supplement: Supplementary file 2 — Additional file 2: Table S2. List of forward and reverse primers used for qRT-PCR analyses [file 12864_2020_6986_MOESM2_ESM.docx]

**TABLE S2 |** List of forward and reverse primers used for qRT-PCR analyses.

| Gene Name | Forward Primer (5'to3') | Reverse Primer (5'to3') |
| --- | --- | --- |
| *GhGRF1* | AATGCTGCTACCTCTGTC | GGTCCTCTATCTCCTCCTA |
| *GhGRF2* | GGTGTTAGCAGGAGGTAG | GGAGACAGTAGGCATCAG |
| *GhGRF3* | TGGGTTTGGAGTTGTTTC | GTGTTGCCGTTACTGTGC |
| *GhGRF4* | AAAGGCACATAAACAGGG | CAGAATGATGGGTAGCAC |
| *GhGRF5* | CCTTTACCCTTATGGCTTCA | GTTCGACGACACCTCCCT |
| *GhGRF6* | CCACTACTACCACCAAGC | TCAGAAGCCACCTGTTTA |
| *GhGRF7* | ACCAACACCCATCTCCTC | ACTCCAGTTCCTGCCATT |
| *GhGRF8* | TGTAACTGCTCGTTTCGG | GCTGATGATGCTCCCTTC |
| *GhGRF9* | ACACCGCCTTCAAATCCC | ACACCTCCCTGGCTCAAC |
| *GhGRF10* | GCTCCTTGAACCTAAACC | GACTGTTCTCGTCGTCTTC |
| *GhGRF11* | TATGGGAAGAAGGTGGAT | ACTGAGGAAACGAGGAGA |
| *GhGRF12* | TGTAGGGATGGTAGCAAT | TCTTCTGAATAGGGAGCA |
| *GhGRF13* | ATCCCACCTCAACTCCTT | ACGGTTCCTGCCTCTATG |
| *GhGRF14* | GCATAGAGGGAAGAACCG | GCTTGAACCCAACAGACA |
| *GhGRF15* | CGTCGGAGAAACCCGTGAA | GCAGAGCCAAAGGAGGAA |
| *GhGRF16* | ATTGAGTGGAGGAGAAGG | TTGAAAGATTGTGAACCC |
| *GhGRF17* | ACTTCGGGATTGGACTTA | GATGATTAGGGACAGGGA |
| *GhUBQ7* | GAAGGCATTCCACCTGACCAAC | CTTGACCTTCTTCTTCTTCTTGTGCTTG |
| *Ghhis3* | GAAGCCTCATCGATACCGTC | CTACCACTACCATCATGG |
| *GaGRF1* | GGAAAGAAATGGCGGTGCTC | GGTAGCACCAGATTGCCAGT |
| *GaGRF2* | CGGGGGTTTTCGTTGTTGTC | ATGAATTGGTGAGGCGGTGT |
| *GaGRF3* | CAGCCTGGTGGCACATCTAA | TCGCCAGAGGAAACAACTCC |
| *GaGRF4* | TCTTTCGCACTCTGGTCCAC | AGCCATCAAAGAACTGGCGA |
| *GaGRF5* | TGCAGCAACTGGTCACGTAT | GTTGATCGATCGGACTGGCT |
| *GaGRF6* | CAGCAGCACAGGTGAGTGTA | GAGGAAGACTGGACAAGGGC |
| *GaGRF7* | AGCAACACGCATCCTCTGAA | ATTGCAACAAAGCTGGCTGG |
| *GaGRF8* | TGGGGAACCTGTGAAACACG | TTCAGTGGGAAACTCACCGT |
| *GaGRF9* | AAATCCGATGGCAACCCCAT | TCCCAAATATCGTGTGGAGAATGA |
| *GaGRF10* | CACCTCTTCCCTCACCAACC | GATGGAAGCTGCTGCTGTTG |
| *GaGRF11* | GACCCAGAACCTGGAAGGTG | GAAGTTGGTGCTGCTGTTGG |
| *GaGRF12* | CCAGGGAGGTGTCGAAGAAC | CCAGGGAGGTGTCGAAGAAC |
| *GaGRF13* | AGCGTTTCAGACAAGTGATTG | GGCTCCCGATAGTGGGTATG |
| *GaGRF14* | TCCCCCACCATTTCTCTGGA | CTTCTGCACCTCCATGGCTC |
| *GaGRF15* | CTTCAAATGCGATAAAGGGTTCT | TGGGTTTTGATGAGGTTGGTT |
| *GaGRF16* | ACGACAGCTCAGTTGCATGA | ACATTCTTACTGCACCGCCA |
| *GaGRF17* | AGGCCAACTGGGTTCACAAA | CGTCTACATCTTCCGGGCTC |
| *GaGRF18* | AAGCCGTCCCAGATCACAAG | CAGCTTTCGAATCACCCCCT |
| *GbGRF1* | TCTAGGGAGACTTCCGTGGG | TGCTCTTGGACTGCTTCCAG |
| *GbGRF2* | CCTGACTGGACTCAACTCTCG | GCGTTGTTCGTCGTGTTGTT |
| *GbGRF3* | AGAAGCTTCAGGGAGCAACC | GCCTCTGGTTTTAGGCCACT |
| *GbGRF4* | TCACCAACACCCATCCTCTTC | CCAAGGTGGCTGGTAATGGG |
| *GbGRF5* | TGGGTTGAAAGAAGTAGATGAACA | CGATGGACTGTCTTTTGGGGT |
| *GbGRF6* | TGGAGGAGTTCGAAACTGGC | AACACCCCATGCATGTTTGC |
| *GbGRF7* | GGAAGGTGTCGCAGAACTGA | TGCCACCAAGCTGCAAATTC |
| *GbGRF8* | TCCACCTCACATCACCTAACTC | CCAAGACGAAAAGCTCCCCA |
| *GbGRF9* | CGCAGCAGCAAGCTAAGATG | GTAGGTGGCTTGGGCTCATT |
| *GbGRF10* | TCAAGAAATGCAACACCACCAC | GGAGTCCATCGCTGTCTAGC |
| *GbGRF11* | GGGTGATTCGTGGAACAAGG | GATAAGGACTGCCGTGGAGG |
| *GbGRF12* | ACAGATCTGGTCACATTTTGAGA | GTAAAGCCTCGGCAAGAGGT |
| *GbGRF13* | ACTTCACCCATTTCGACCCC | GCCCCTGCTTTGAACCATTG |
| *GbGRF14* | TTCTAATGTGGCAGTGCCGT | AACAGCATCCTGAGAGCACC |
| *GbGRF15* | ATTCTGGTAGGGGTGGTGGG | GGGAACTTGGATTCCACCCG |
| *GbGRF16* | GCCTCCTATGCATCCTCCAC | ACATTGCTGTGCCATCTTGC |
| *GbGRF17* | TGGACTCCAACGCCATTACA | GGTTCAGGTTCAGCATTGGC |
| *GbGRF18* | AATCAAGTGCTTCCCAGGCA | GCATCTGGGTAGAAGCTCCC |
| *GbGRF19* | TGGTGGTCTCCATGCCAATC | AATGGAGAGGTTGGTGCCAG |
| *GbGRF20* | AATCAGAAACCATCCAGGGG | GGGATAAGGAGTTCAGCAGGG |
| *GbGRF21* | GCCATGGCAAGGTTTTCTGG | CAACAAACTGTGGGCGAGTG |
| *GbGRF22* | TCTGAGGCTTTGGGAAGCAA | GTGTCCCTGTCCCTGGTTTT |
| *GbGRF23* | CCAGGGAGGTGTCGAAGAAC | CCAACAGACAAAGGGTGGGT |
