# Supplementary figures and images for: Genome-wide characterization of the GRF family and their roles in response to salt stress in Gossypium
Source: BMC Genomics. 2020 Aug 24;21:575. doi: 10.1186/s12864-020-06986-0 (PMC7444260; doi:10.1186/s12864-020-06986-0)

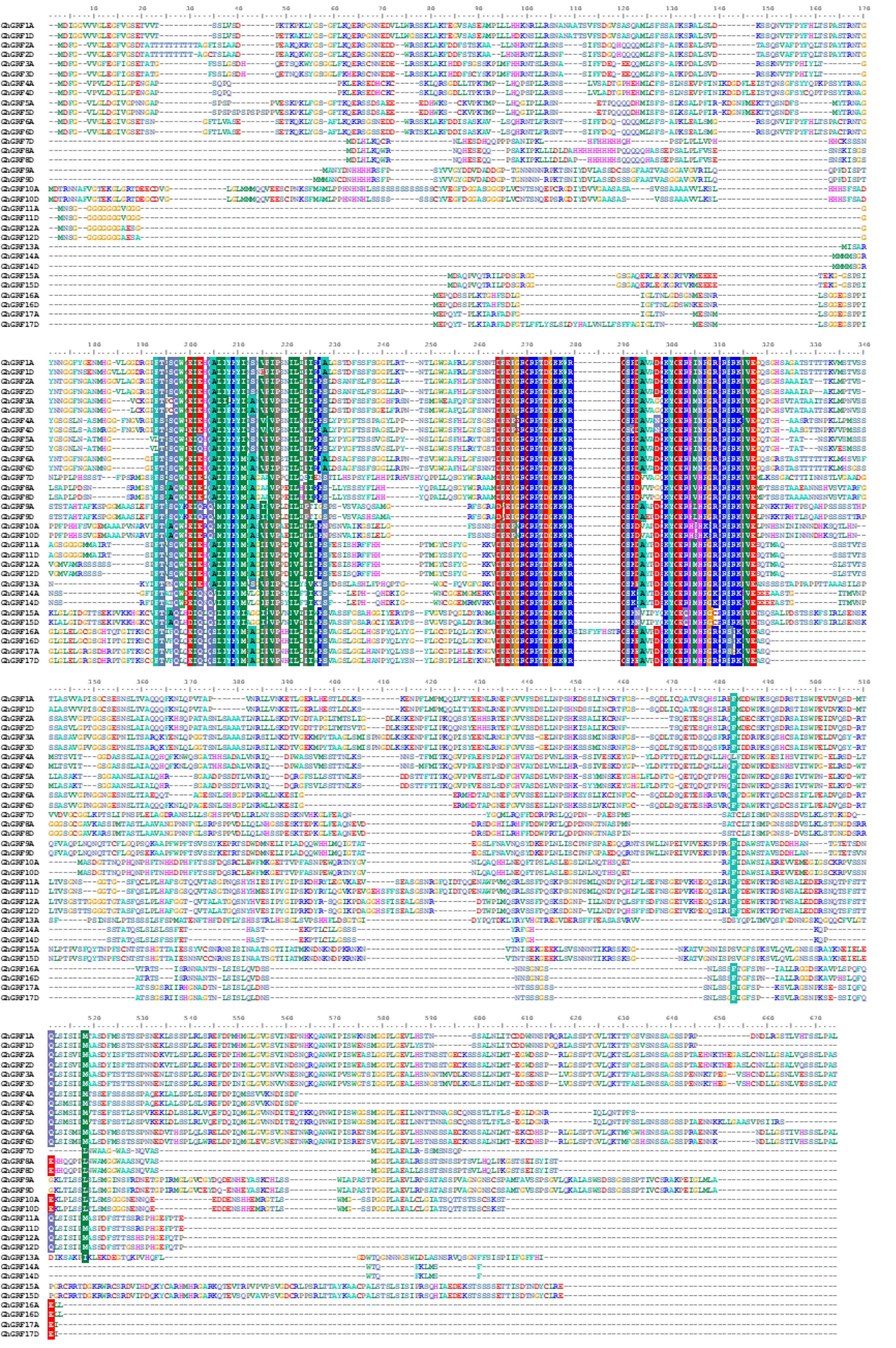

Supplement: Supplementary file 3 — Additional file 3: Figure S1. Multiple alignment of GhGRF (Gossypium hirsutum GRF) protein sequences [file 12864_2020_6986_MOESM3_ESM.tif]

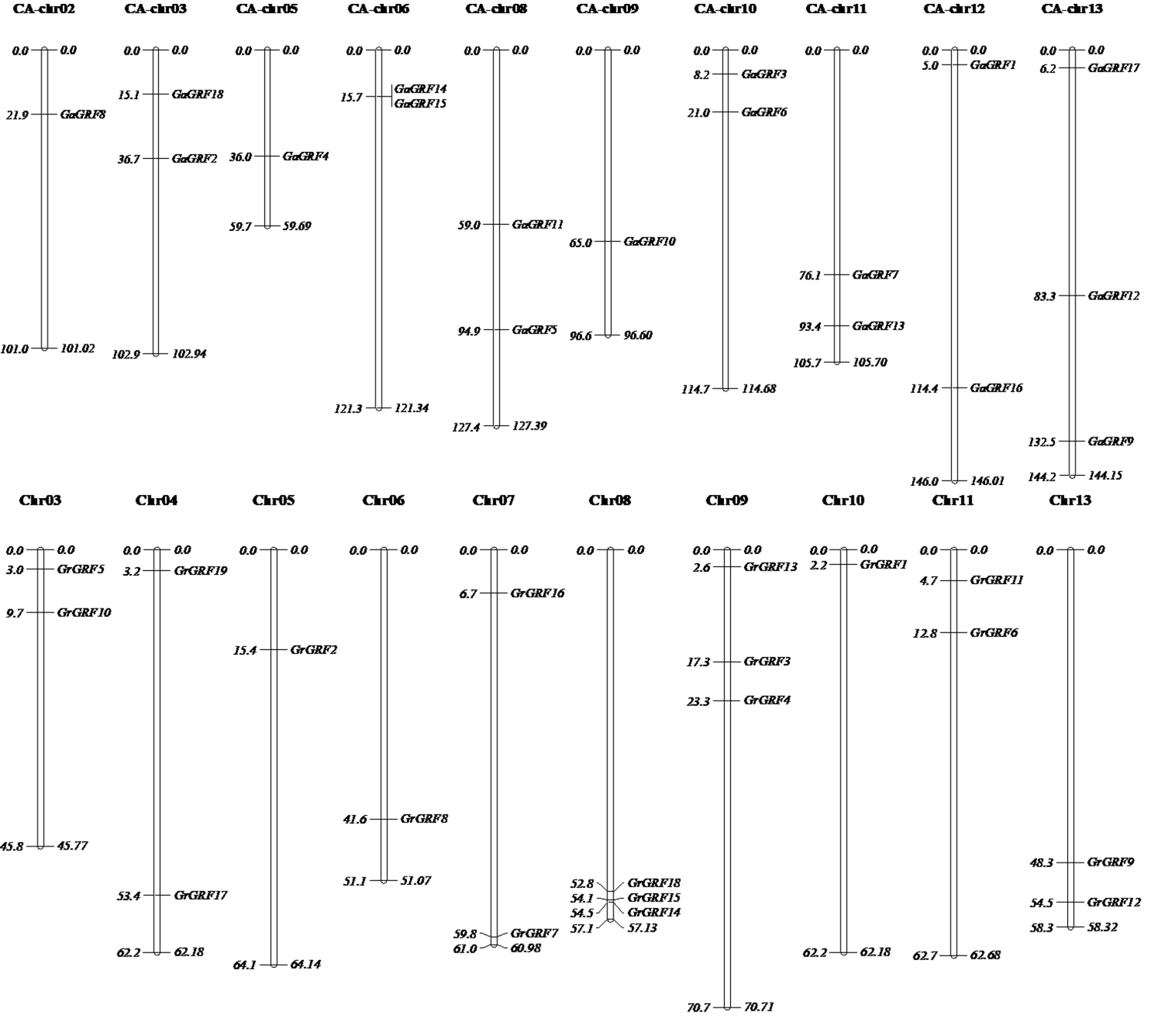

Supplement: Supplementary file 4 — Additional file 4: Figure S2. Chromosome distribution of GaGRF (Gossypium arboreum GRF) and GrGRF (Gossypium raimondii GRF) genes. [file 12864_2020_6986_MOESM4_ESM.tif]

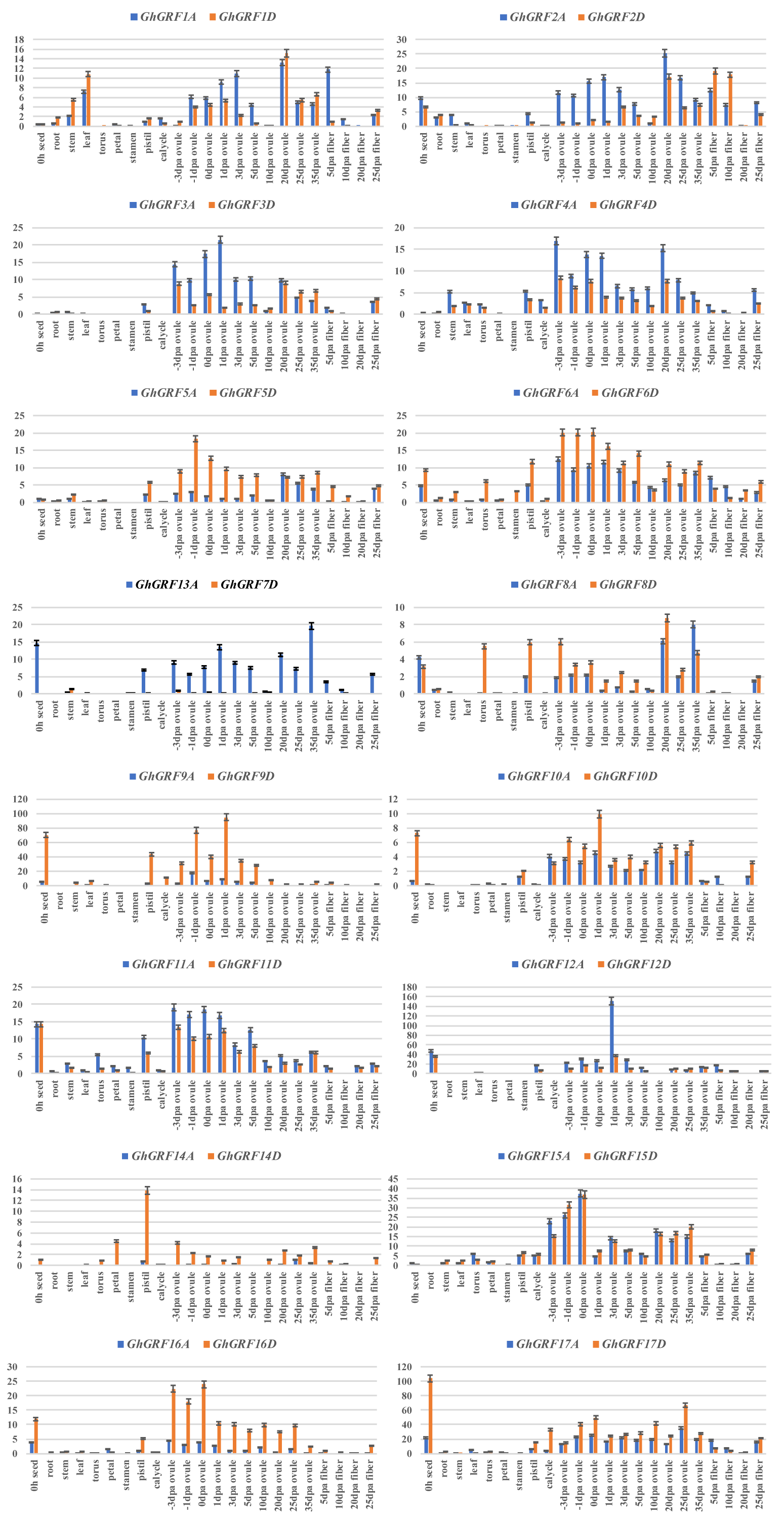

Supplement: Supplementary file 5 — Additional file 5: Figure S3. Expression patterns of GRF genes in G. hirsutum using RNA-seq data. The RNA-seq data expression profiles were from Zhang et al., (2015) and ccNET database [46]. FPKM represents fragments per kilobase of exon model per million mapped reads. DPA, days post anthesis [file 12864_2020_6986_MOESM5_ESM.tif]
